# Green Antimicrobials

**DOI:** 10.3390/antibiotics12071128

**Published:** 2023-06-29

**Authors:** Helena P. Felgueiras

**Affiliations:** Centre for Textile Science and Technology (2C2T), University of Minho, Campus de Azurém, 4800-058 Guimarães, Portugal; helena.felgueiras@2c2t.uminho.pt

In the last couple of years, the awareness of climate change and high pollution levels have raised our sense of ecological responsibility. Pharmaceutical industries play a major role in these issues; as such, alternatives must be found. New environmentally friendly approaches to deal with the growing concern associated with antimicrobial-resistant bacteria are also in great demand. The excessive consumption and misuse of these agents have accelerated the rise of such pathogens responsible for compromising global health—not only the health of humans, but also the health of all living systems. Considering our natural resources are in great danger, finding green and less environmentally impactful alternatives for fighting these resistant microbials is imperative. From green chemistries, natural extracts and waste products, the sources of these alternate antimicrobial agents can be immense, and their implications are of great impact for future generations [1,2,3].

Fernández-Fernández et al. reported the great biodiversity and biomass of microorganisms found in soil microbiota that could be used in the production of new antimicrobial agents. Through the MicroMundo project, they illustrated the relevant link between science and education and the benefits of implementing service-learning methodologies to raise awareness of the antimicrobial resistance problem. They collected soil samples from different areas and analyzed their content for antimicrobial-producing bacteria. In total, 132 potentially producing bacteria were identified, from which 18 isolates were deemed low producers of antimicrobial agents, 12 were recognized as medium producers, and 2 were considered highly antimicrobial-producing isolates [4]. Aside from soil, agricultural wastes can also serve as sources of antimicrobial agents. Arumugam et al. explored the abilities of crude solvent extracts of both groundnut shells and black gram pods to reduce aerolysin formation and biofilm matrix formation by *Aeromonas hydrophila*, an opportunistic bacterium, responsible for many diseases in humans and animals, particularly aquatic species. Twelve potent metabolites extracted from these wastes showed interactions with aerolysin during molecular docking analysis, confirming their potential for uses in pharmacological solutions for treating *A. hydrophila*-induced infections in aquaculture [5].

Foodborne pathogens can pose great risks to human health. Torres Neto et al. investigated the antimicrobial potency of the essential oils of oregano, thyme, lemongrass and their blends against three foodborne pathogens, *Salmonella enterica* serotype Enteritidis, *Escherichia coli* and *Staphylococcus aureus*. The data highlighted the ability of the blends containing thyme to inhibit and kill each bacterium individually [6]. Adnan et al. focused their efforts on finding effective agents against the foodborne pathogen *Listeria monocytogenes*, responsible for the disease listeriosis. Here, active biosurfactants and their potential targets were tested against the bacterium, revealing their ability in regulating several bacterium pathways responsible for the bacterium’s pathogenic nature, evidencing their pharmacological potency [7]. Stavropoulou et al. demonstrated that honey samples obtained from different geographical locations in Greece and with diverse pollen origins were influenced by the pollinizing animals. In fact, they detected 335 distinct taxa in honey microbiota, native to the gut microbiota of melliferous bees and microbiota of their flowering plants, including beneficial bacteria like probiotic strains. These could work as indicators of the authenticity of honey, facilitating the identification of honey samples with potent antimicrobial profiles [8]. β-Glucan is an important natural prebiotic abundant in lentinan, cereals and the yeast cell wall. Zhou et al. evaluated the influence of this natural-origin component on growth performance and intestinal epithelium functions and established β-glucan as an attenuator of intestinal damage by suppressing the secretion of inflammatory cytokines, enhancing serum immunoglobulins and improving intestinal epithelium functions and microbiota [9].

Skin disorders have benefited immensely from natural-origin antimicrobials. Diabetic foot ulcers, for instance, have been shown to accelerate their healing in the presence of antimicrobial peptides derived from green, eco-friendly processes [10]. In chronic wound care, many antiseptic agents have also been highlighted, namely octenidine and polyhexamide, as beneficial for wound care without inducing any cytotoxic effects or raising significant environmental concerns [11]. Ranjutha et al. identified synergisms between ceftriaxone and *Polyalthia longifolia* methanol leaf extract against methicillin-resistant *Staphylococcus aureus*, supporting their use as an etiological agent for skin disease therapies. The gene responsible for the bacterium resistance was significantly suppressed by the antimicrobial agents’ combination, and the same occurred with the concentration required to eliminate the bacterium [12]. Matias et al. disclosed the seaweed *Gelidium corneum* as a sustainable source of antimicrobial ingredients for new dermatological formulations, highlighting its potential to be explored in a circular economy context. For this purpose, seaweed fractions were compiled and examined against common pathogenic bacteria. The data showed that the antimicrobial effects of the fractions were manifested in the form of membrane hyperpolarization and DNA damage [13].

Innovations in silver nanoparticle production have also taken advantage of green processes. For instance, Padmanabhan et al. reported the formation of clusters containing biphasic calcium phosphate-modified silver nanoparticles for improved antimicrobial and cytocompatibility performances and, thus, engineering of bone tissue replacements [14]. Sharma et al. explored the use of silver nanoparticles embedded into polymeric films to prevent and mitigate pathogen transmission in biomedical surfaces. It was shown that the incorporation of silver nanoparticles inhibited the growth of Gram-positive and Gram-negative bacteria, generating a polymeric substrate that is both cost-effective and highly scalable, by means of simple production methodologies (sputter deposition) [15].
